# Women Doctors and the London County Council

**Published:** 1914-04-18

**Authors:** 


### THE EDITOR'S LETTER-BOX.

### Women Doctors and the London County Council.

*To the Editor of* The Hospital.

Sir,—Without any intention to offer my opinion on the large question which is raised in the letters on this subject in your issue of April 11, may I just demur to two or three points in them? First, Dr. Bone says "the woman herself should be the judge." Of what? Of whether if she marries she will have children, or of whether maternity interferes with her public health work? Of the latter question she is not the best judge, and if she answers the former in the negative, she is not the sort of woman (in my opinion, *bien entendu*) that any public authority should employ. The statement that the employer can dismiss his married (female) employee if he is dissatisfied shirks part of the issue; for how is the employer to know whether his employee is or is not neglecting her duties to her family by continuing in his employment?

Then your other correspondent thinks the County Council, by its regulations, treats marriage as disgraceful. Not at all; it only treats marriage and a certain occupation as incompatible. It may, and probably does, regard marriage as infinitely the higher and more honourable calling—estate, if you object to calling—of the two. Nor is the root of the matter that the Council regards a mother as necessarily in continuous ill-health. It merely, so I judge, regards the enforced absence from work which is desirable and often inevitable throughout pregnancy and lactation as prejudicial to the efficiency of medical inspection in the hands of those who are likely to bear children.

I hold no brief for the Council, so beg to remain,

Yours faithfully, Interested.

[Our correspondent will not fail to note the leading article on first page of this issue.—Ed., The Hospital.]

